# Subchondral bone density distribution of the talus in clinically normal Labrador Retrievers

**DOI:** 10.1186/s12917-016-0678-8

**Published:** 2016-03-15

**Authors:** W. Dingemanse, M. Müller-Gerbl, I. Jonkers, J. Vander Sloten, H. van Bree, I. Gielen

**Affiliations:** Department of Medical Imaging of Domestic Animals and Orthopaedics of Small Animals Faculty of Veterinary Medicine, Ghent University, Merelbeke, Belgium; Institute of Anatomy, Basel University, Basel, Switzerland; Human Movement Biomechanics Research Group, Kinesiology Department, KU Leuven, Leuven, Belgium; Biomechanics Section, Faculty of Engineering Science, KU Leuven, Leuven, Belgium

**Keywords:** CTOAM, Dog, Joint loading, Osteochondrosis, Subchondral bone, Talus

## Abstract

**Background:**

Bones continually adapt their morphology to their load bearing function. At the level of the subchondral bone, the density distribution is highly correlated with the loading distribution of the joint. Therefore, subchondral bone density distribution can be used to study joint biomechanics non-invasively. In addition physiological and pathological joint loading is an important aspect of orthopaedic disease, and research focusing on joint biomechanics will benefit veterinary orthopaedics. This study was conducted to evaluate density distribution in the subchondral bone of the canine talus, as a parameter reflecting the long-term joint loading in the tarsocrural joint.

**Results:**

Two main density maxima were found, one proximally on the medial trochlear ridge and one distally on the lateral trochlear ridge. All joints showed very similar density distribution patterns and no significant differences were found in the localisation of the density maxima between left and right limbs and between dogs.

**Conclusions:**

Based on the density distribution the lateral trochlear ridge is most likely subjected to highest loads within the tarsocrural joint. The joint loading distribution is very similar between dogs of the same breed. In addition, the joint loading distribution supports previous suggestions of the important role of biomechanics in the development of OC lesions in the tarsus. Important benefits of computed tomographic osteoabsorptiometry (CTOAM), i.e. the possibility of in vivo imaging and temporal evaluation, make this technique a valuable addition to the field of veterinary orthopaedic research.

## Background

Joint loading, including tensile and compressive stresses, is an important factor in cartilage and subchondral bone physiology and pathology [1–3]. Changes in loading and biomechanical properties of these important load-bearing structures, play a key-role in the development and progression of orthopaedic disease [4].

In recent years, different methods have been applied to study joint biomechanics both in vitro and in vivo. The initial studies presented detailed anatomical descriptions of joint structures [5, 6], followed by studies describing pressure distributions and contact areas [7, 8]. These studies were often done on cadaveric specimens and required a certain degree of dissection, thus altering joint kinematics. In vivo biomechanics are often limited to kinetic and kinematic research using marker data and pressure plates [4]. Actual joint loading cannot easily be assessed non-invasively in vivo, since it requires intra-articular insertion of pressure films [9] making it difficult to apply in studies using larger populations and patient populations. Subchondral bone density is directly influenced by joint biomechanics and limb function and can be used to evaluate joint biomechanics.

The stresses acting on the joint surface induce modelling and remodelling of the bony tissue, depending on whether the local strains either exceed the modelling threshold or stay below the remodelling threshold. Because of that, increased joint loading leads to increased local strains and bone modelling ensures an increase in subchondral bone density to withstand the increased loading [1, 3]. In addition, altered joint biomechanics lead to altered joint loading distribution, leading in turn to alterations in the subchondral bone density distribution [10, 11].

The subchondral bone density in joints is highly correlated with joint loading and reflects the loading history of the joint [3, 11–13]. Using computer tomographic osteoabsorptiometry (CTOAM), the density distribution of the subchondral bone can be visualised and evaluated [3, 11–13]. In order to evaluate subchondral bone density and changes associated with orthopaedic conditions, the normal, physiological subchondral bone density distribution has to be described first.

In addition, this type of biomechanical research can help to elucidate the role of joint biomechanics in the development of osteochondrosis (OC) [14]. Osteochondrosis is an orthopaedic condition in dogs that is considered to be multifactorial, with hereditary, dietary and environmental factors playing a role [15]. An environmental factor likely to influence the occurrence of OC is joint biomechanics, since OC lesions are often found in specific locations within the joint [16–19]. In the tarsocrural joint, lesions can be found medially (medial trochlear ridge tarsocrural osteochondrosis (MTRT-OC)) and laterally (lateral trochlear ridge tarsocrural osteochondrosis (LTRT-OC)) [14, 20, 21]. The specific joint anatomy likely affects the location of the OC lesions, and this study can aid in the understanding of the pathophysiology of this condition.

This study was conducted to describe the subchondral bone density distribution of the talus of healthy Labrador Retrievers non-invasively, as a parameter reflecting long-term joint loading in the tarsocrural joint, using CTOAM. The authors hypothesise an inhomogeneous distribution of the density of the subchondral bone.

## Methods

### Study population

A total of 20 tarsal joints (ten left and ten right) from ten adult (age 24–28 months) Labrador Retrievers, submitted for computer tomographic (CT) examination of the elbow joint for the screening of elbow dysplasia, were included in this study. The study was approved by the ethical committee of the Faculty of Veterinary Medicine, Ghent University (approval nr. EC2011/193) and informed, written owner consent was obtained in each case. Inclusion criteria for this study were no abnormalities on orthopaedic examination and lameness evaluation and no abnormalities on radiographs of hips, elbows and tarsal joints. After CT examination of the elbow joint, the tarsal joints were scanned as well.

### Image acquisition

Under general anaesthesia and the dog positioned in ventral recumbency, CT images were acquired from the tarsal joints using a four slice helical CT scanner (Lightspeed Qx/i, General Electric Medical Systems, Milwaukee, WI, USA). The CT parameters were 120 kVp and 300 mAs. Contiguous, 1,25 mm collimated, transverse images were obtained in a soft tissue reconstruction algorithm. Left and right tarsal joints were scanned simultaneously, with the tarsal joints in extension, according to patient protocol [21]. Acquisition time was approximately five minutes, including repositioning after CT examination of the elbow joints.

### Image analysis

**Fig. 1:**
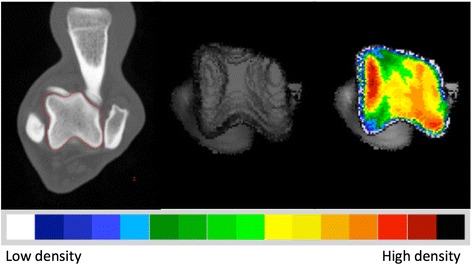
CTOAM workflow in Analyze. From left to right: segmentation of the talus; 3D rendering of the proximal view; overlay of the false-colour map of the subchondral bone density distribution

**Fig. 2:**
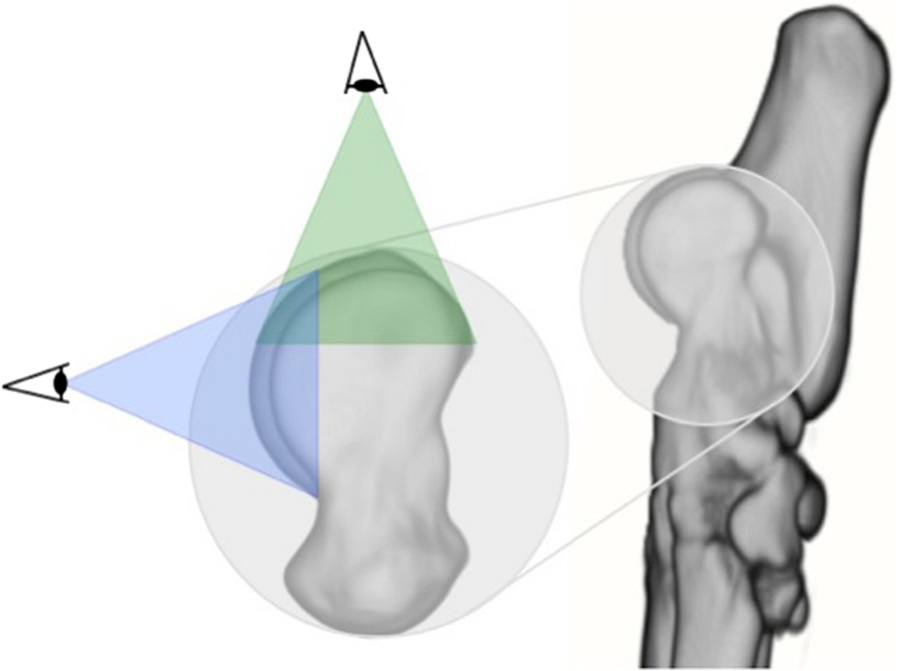
Three dimensional reconstruction of the tarsal and metatarsal bones of the right foot, medial view. Line of sight for the two 3D reconstructions, proximal view (green) and distal view (blue). The use of these two views provides full visualisation of the trochlear ridges

**Fig. 3:**
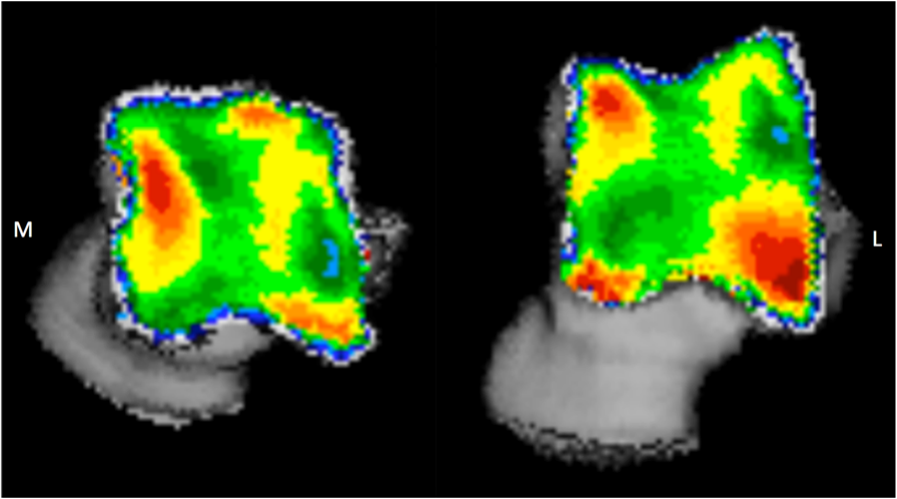
Typical subchondral bone density distribution of the talus. Left talus in a proximal (left) and distal (right) view

The CT images were exported in DICOM format to commercially available software (Analyze 11.0, Biomedical Imaging Resource, Mayo Foundation, Rochester, MN, USA), used to complete the CTOAM workflow (Fig. 1). In the first step, the talus was segmented using the segmentation algorithm in ‘Analyze’. Based on the segmented images, two different three-dimensional (3D) views of the trochlear ridges were reconstructed (Fig. 2). A proximal view was reconstructed first, and the distal view was obtained by tilting the proximal view backwards approximately 90 degrees. This allowed the evaluation of the entire proximal talar joint surface of the lateral and medial trochlear ridge (Fig. 3). Subsequently, the subchondral bone plate of the articulating surface was isolated and reconstructed in exactly the same orientations. The maximum bone density was projected onto the articular surface using a maximum intensity projection (MIP). With a MIP, the three-dimensional (3D) data volume (in voxels) of the subchondral bone plate is converted to a 2D image (in pixels) in which each pixel represents the maximum value (based on the HU). This maximum value is obtained from the voxels along the line perpendicular to the pixel in the 2D image. The length of this line, i.e. the depth of the MIP, is based on the thickness of the subchondral bone plate and was set at 1.5 mm. This MIP view was then converted to a false colour scale, where the range of 200 – 1200 Hounsfield Units (HU) was divided in value ranges of 100 HU each representing a colour. In descending order these colours were black, dark red, light red, orange, yellow, dark green, light green, dark blue, light blue, and white. This resulted in a densitogram (Figs. 1 and 3), which displays lines of isodensities, i.e. lines connecting regions of equal density. This densitogram is a visual representation of the apparent density distribution and was further evaluated.

**Fig. 4:**
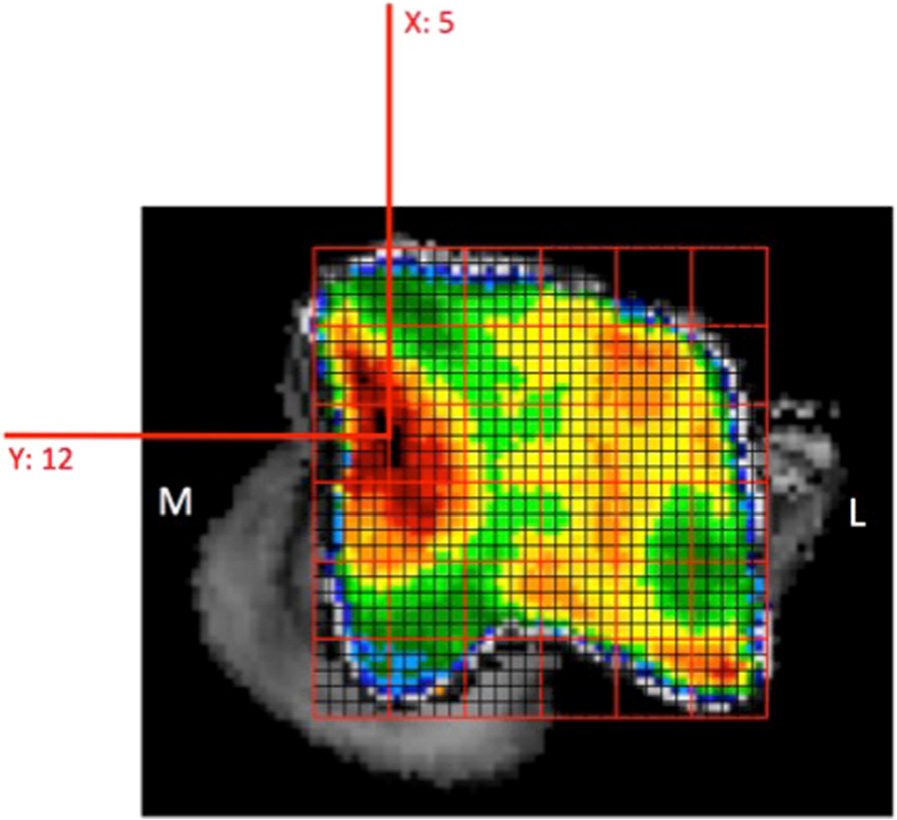
Positioning of the grid on a proximal view of the talus and description of the subchondral bone density maximum by x- and y-coordinates

For quantification purposes, the density values (in HU) were converted to 8-bit values, i.e. 256 density values, which were split equally over eight bins, according to literature [22]. Thus, each bin contains a range of 32 density values. A density maximum was defined as an area with density values in the two highest density bins of the densitogram. To allow the comparison of the individual subchondral bone density distributions, a 30 × 30 unit grid was projected over the densitogram of the proximal and dorsal view of the trochlear ridges. The grid edges were positioned thus the entire joint surface could fit within. The number of units in each grid was kept the same, to standardize the coordinates of the density maxima. The x- and y-coordinates (Fig. 4) were used to describe the location of the density maxima on the joint surface.

In addition, the size of the maximum was described as a ratio of the area of the density maximum and the joint surface area of the proximal and distal view respectively, and defined as the maximum area ratio (MAR).

$$ \mathrm{M}\mathrm{A}\mathrm{R} = \mathrm{number}\ \mathrm{of}\ \mathrm{pixels}\ \mathrm{of}\ \mathrm{the}\ \mathrm{density}\ \mathrm{maximum}\ /\ \mathrm{number}\ \mathrm{of}\ \mathrm{pixels}\ \mathrm{of}\ \mathrm{the}\ \mathrm{total}\ \mathrm{joint}\ \mathrm{surface} $$

The use of MAR allows a relative comparison between individuals, and accounts for size-differences.

### Statistics

Using commercially available software (SPSS Statistics 22), the location of the density maxima and the MAR were compared between left and right and between dogs. Data was evaluated using a Student’s *T*-test and ANOVA (with Bonferroni post-hoc) and significance was set at *P* < .05.

## Results

### Regional variation of subchondral bone density

The proximal and distal reconstructions provided full visualization of the subchondral bone, with a small visual overlap in the transitional area (proximodorsal area) (Fig. 2). The subchondral bone density distribution showed considerable regional differences in both the medial and the lateral trochlear ridge. Hounsfield Units generally ranged from 200–1200, although in some dogs (*n* = 2) the upper range was limited to around 1000 HU.

### Differences in subchondral bone density distribution between medial and lateral trochlear ridge

In general, the lateral trochlear ridge had a higher apparent density in comparison to the medial trochlear ridge, as illustrated by the colour map (Fig. 3). The medial and lateral trochlear ridges showed a distinctly different density pattern.

The medial trochlear ridge had a density maximum in its proximal part. More distally, density values were lower. In 80 % of the joints (*n* = 16), a focal additional density maximum was present at the most distal part of the trochlear ridge.

On the lateral trochlear ridge, the density maximum was found at the distal part of the trochlear ridge at the level where the medial trochlear ridge shows an area of low density. This density maximum was larger (Table 1) and showed a larger variety in shape than the maximum on the medial trochlear ridge. The density maxima on medial and lateral trochlear ridges were located adjacent to the medial and lateral border of the ridge respectively.

**Table 1:** Results summary

		Left	Right	*P*-value
Proximal view	Total # pixels	2254.8 (482.1)	2226.4 (494.9)	.909
	Area max # pixels	737.9 (212.4)	716.5 (218.0)	.845
	MAR	32.4 (4.3)	31.6 (3.9)	.720
Distal view	Total # pixels	2348.1 (404.2)	2288.8 (366.4)	.763
	Area max # pixels	919.9 (184.4)	944.4 (208.5)	.807
	MAR	39.5 (5.0)	41.5 (6.6)	.495

Total number of pixels, number of pixels of the density maximum and MAR. Values displayed as mean (SD)

### Quantification of density maxima

**Fig. 5:**
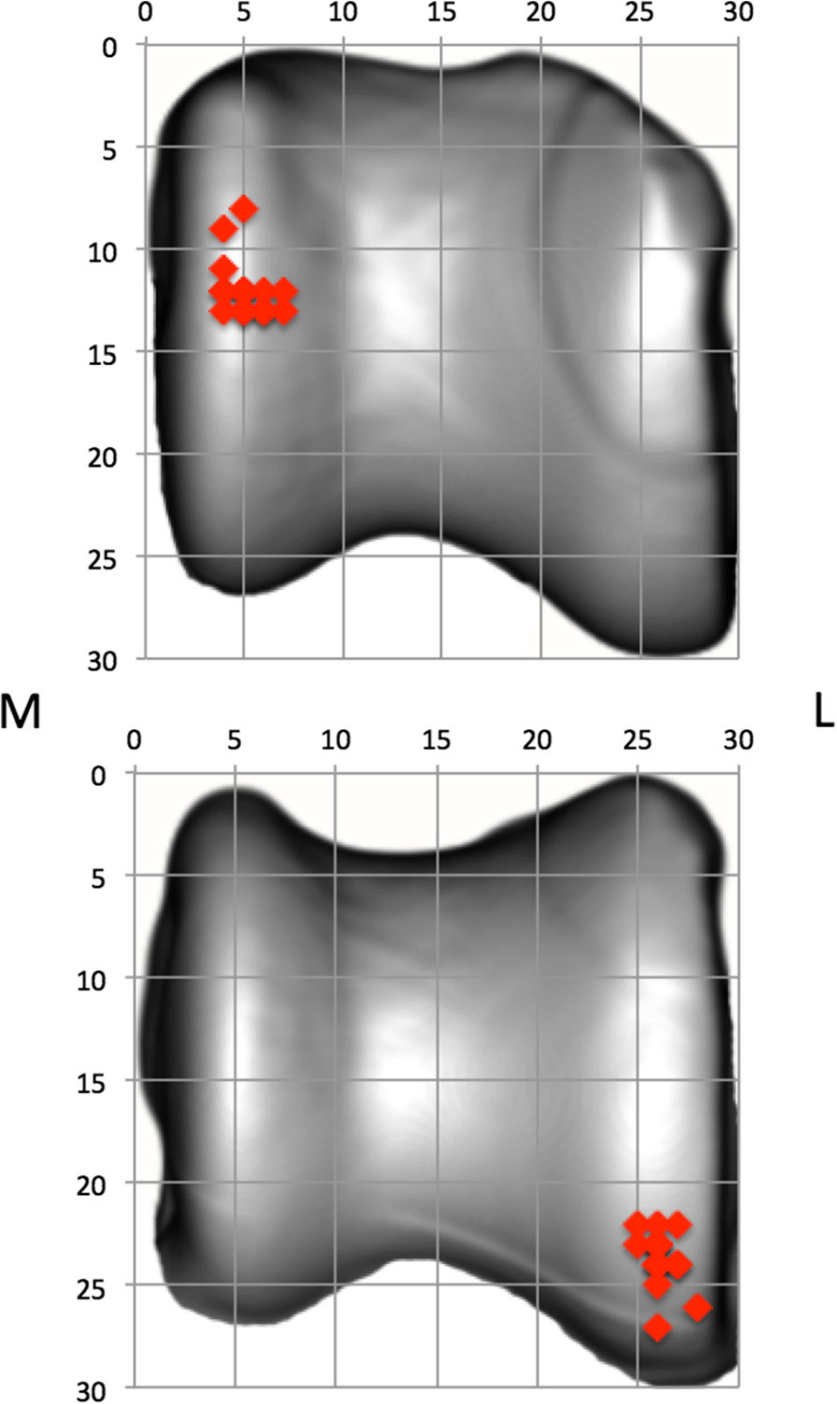
Summation view of the density maxima coordinates. Proximal (top) and distal (bottom) view, right coordinates were mirrored to left

The location of the density maxima on the standardized grid is displayed in a summary view for both views of the talus (Fig. 5). The density maxima clearly display a very similar distribution in all dogs. No significant differences in the coordinates were found between left and right joints (*p*-values .607 and .540) and between different dogs (*p* values .755 and .367).

### Comparison of MAR

There was no significant difference in the MAR between left and right (Table 1). Between dogs, there was no significant difference for the MAR in the proximal view (*p*-value .505), but a significant difference was found in the distal view (*p*-value .003).

## Discussion

This study describes the subchondral bone density distribution of the talus in a group of healthy Labrador Retrievers, using conventional CT data and CTOAM. In addition to a description and visual representation of the subchondral bone density distribution. Density maxima were described using a standardised grid overlay and the maximum area ratio (MAR) was calculated.

Previous studies in humans have shown regional subchondral bone density variations in many different joints [11, 12, 23], but studies in dogs have been limited to the elbow and stifle joint [24–26]. In this study, considerable regional differences of subchondral bone density were found in the convex surface of the talus, articulating with the distal tibia.

The density distribution of the trochlear ridges of the proximal talus is characterized by two density maxima. One is located at the proximal part of the medial trochlear ridge and the other one is located more distally on the lateral trochlear ridge. In addition, the apparent density of the lateral trochlear ridge is higher than the apparent density of the medial trochlear ridge. A possible explanation for this is the fact that the lateral trochlear ridge in the dog is more pronounced and is more likely to endure increased loads during gait. Geometry plays a major role in the development of subchondral bone density patterns, as it determines the magnitude and direction of the dynamic loads, which in turn will guide the modelling process, leading to morphological adaptations [3, 27], which is in this case an increase in apparent density.

Both the location of the density maxima and the MAR showed no significant differences between left and right limbs. A recent study described asymmetry in limb and joint mechanics in orthopedically sound Labrador Retrievers [28]. Mechanical dominance has been described in various species, and in dogs right hind limb dominance appears to be most common [29]. These conclusions are based on the calculation of the total support moment of the limbs, and showed that the tarsal joint moment was significantly larger on the dominant side. Mechanical dominance was not evaluated using gait analysis in the dogs used in this study. Based on our findings, we assume that the dogs used in this study have symmetrical gait, or that the differences in case of asymmetry due to hind limb dominance, did not significantly effect subchondral bone density distribution. Whether or not hind limb dominance in dogs has an influence on subchondral bone density is a very interesting topic, and is subject of further research.

On the proximal view there was no significant difference found for the MAR between dogs, whereas on the distal view there was a significant difference for the MAR. As mentioned above, the maximum on the lateral trochlear ridge was located distally, so it was visualised best on the distal view, and showed more variety in shape compared to the maximum on the medial trochlear ridge. This explains the difference in MAR between dogs in the distal view. A possible explanation is that the proximal part of the medial trochlear ridge is subjected to more homogeneous loading. Another possibility, that is likely to play a simultaneous role, is that the force-transmitting area of the medial trochlear ridge is much more constant between dogs, whereas for the lateral trochlear ridge this can vary more between dogs.

Possible drawback of CTOAM for the evaluation of subchondral bone density is that the density distribution of a 3D volume (the voxels) is displayed in 2D (pixels). Because the density is evaluated over the thickness of the subchondral bone plate, perpendicular to the line of sight on the joint surface, this will cause no problems on flat articular surfaces. On more curved articular surfaces, the use of multiple views is necessary to evaluate the subchondral bone density distribution.

Differences in the size of the area of maximum density can be caused by absolute size differences (i.e. larger or smaller talus) but in this study this effect will be very minimal since all dogs were Labrador Retrievers of approximately the same size, weight, and age. Another reason is differences in scanning parameters, specifically the size of the field of view (FOV). Pixel size depends on the size of the scanned object and FOV used for the scan. Since we used consistently a FOV of 512×512, this effect will be minimal due to a standardised position of the joint, and minimal size differences between the dogs used in this study. The use of the MAR, allows a relative comparison of the area of maximum density, accounting for the above confounders when using absolute size values.

When considering joint loading and joint congruency, another important factor is the joint cartilage. Joint cartilage has the important biomechanical role to provide an even distribution of the joint loading on the articular surface [30]. Thicker cartilage is found in places with higher biomechanical loads. A study by Brunnberg et al. supports our conclusion that the lateral trochlear ridge is most likely subjected to higher loads. The cartilage of the lateral trochlear ridge is significantly thicker than the cartilage at the medial trochlear ridge [31].

The location of the density maximum on the medial trochlear ridge is the same location where the majority of MTRT-OC lesions are found [14]. Repetitive loading above the bone modelling threshold, can cause accumulation of microdamage to the bone [32]. Areas with increased subchondral bone density, and thus increased loading conditions, are more likely to be subjected to loading causing microdamage as well. On the talus, these areas subjected to high loading coincide with the location of MTRT-OC lesions. Thus, this study supports previous suggestions that repetitive microdamage [33] is an important factor in the development of OC, although more research is necessary to elucidate the exact pathophysiology.

Lesions on the lateral trochlear ridge (LTRT-OC lesions), are larger than MTRT-OC lesions and have a larger variation in size [14]. Interestingly, the subchondral bone density maximum on the lateral trochlear ridge is larger and shows more variation compared to the medial trochlear ridge, and similar distribution as the OC lesions.

However, changes in subchondral bone density can be cause or effect of OC lesions. A local increase in subchondral bone density, as is the case at the level of a subchondral bone density maximum, may increase the discrepancy between the biomechanical properties of two articulating surfaces. In humans, this mismatch has been suggested to contribute to the development of OC lesions [33, 34].

## Conclusion

This study shows a distinct pattern of subchondral bone density in the talus of healthy, adult Labrador Retrievers. This pattern, or density distribution, provides more information on the biomechanical aspects of the tarsocrural joint and the morphological adaptations under normal joint loading conditions. The influence of altered joint kinematics, bone geometry and leg conformation on the subchondral bone density distribution remains subject of further research.

Although the evaluation of the subchondral bone density distribution pattern supports previous suggestions on the role of joint biomechanics in the development of tarsocrural OC, more research is needed to determine cause and effect. Therefore, research should focus on early stages of OC lesions and systematically review all factors contributing to the biomechanical joint loading.

In the field of veterinary biomechanics, CTOAM could provide new insights in physiological joint loading distribution, and alterations in pathological conditions. The technique can be used in vivo, in patient populations, and to evaluate temporal changes, for instance following orthopaedic surgery. This implies significant advantages compared to more traditional and invasive techniques used to evaluate joint loading.

## Abbreviations

3D: three-dimensional
CT: computed tomographic
CTOAM: computed tomographic osteoabsorptiometry
HU: hounsfield units
LTRT-OC: lateral trochlear ridge tarsocrural osteochondrosis
MAR: maximum area ratio
MIP: maximum intensity projection
MTRT-OC: medial trochlear ridge tarsocrural osteochondrosis
OC: osteochondrosis
